# An automated liver segmentation in liver iron concentration map using fuzzy c-means clustering combined with anatomical landmark data

**DOI:** 10.1186/s12880-021-00669-2

**Published:** 2021-09-28

**Authors:** Kittichai Wantanajittikul, Pairash Saiviroonporn, Suwit Saekho, Rungroj Krittayaphong, Vip Viprakasit

**Affiliations:** 1 Department of Radiologic Technology, Faculty of Associated Medical Sciences, Chiang Mai University, Chiang Mai, Thailand; 2 Division of Diagnostic Radiology, Department of Radiology, Faculty of Medicine, Siriraj Hospital, Mahidol University, Bangkok, 10700 Thailand; 3 Division of Cardiology, Department of Medicine, Siriraj Hospital, Mahidol University, Bangkok, Thailand; 4 Haematology/Oncology Division, Department of Pediatrics and Thalassemia Center, Siriraj Hospital, Mahidol University, Bangkok, Thailand

**Keywords:** Magnetic resonance image (MRI), Liver segmentation, Liver iron concentration (LIC), Fuzzy c-means (FCM) clustering, Anatomical landmark data

## Abstract

**Background:**

To estimate median liver iron concentration (LIC) calculated from magnetic resonance imaging, excluded vessels of the liver parenchyma region were defined manually. Previous works proposed the automated method for excluding vessels from the liver region. However, only user-defined liver region remained a manual process. Therefore, this work aimed to develop an automated liver region segmentation technique to automate the whole process of median LIC calculation.

**Methods:**

553 MR examinations from 471 thalassemia major patients were used in this study. LIC maps (in mg/g dry weight) were calculated and used as the input of segmentation procedures. Anatomical landmark data were detected and used to restrict ROI. After that, the liver region was segmented using fuzzy c-means clustering and reduced segmentation errors by morphological processes. According to the clinical application, erosion with a suitable size of the structuring element was applied to reduce the segmented liver region to avoid uncertainty around the edge of the liver. The segmentation results were evaluated by comparing with manual segmentation performed by a board-certified radiologist.

**Results:**

The proposed method was able to produce a good grade output in approximately 81% of all data. Approximately 11% of all data required an easy modification step. The rest of the output, approximately 8%, was an unsuccessful grade and required manual intervention by a user. For the evaluation matrices, percent dice similarity coefficient (*%DSC*) was in the range 86–92, percent Jaccard index (*%JC*) was 78–86, and Hausdorff distance (*H*) was 14–28 mm, respectively. In this study, percent false positive (*%FP*) and percent false negative (*%FN*) were applied to evaluate under- and over-segmentation that other evaluation matrices could not handle. The average of operation times could be reduced from 10 s per case using traditional method, to 1.5 s per case using our proposed method.

**Conclusion:**

The experimental results showed that the proposed method provided an effective automated liver segmentation technique, which can be applied clinically for automated median LIC calculation in thalassemia major patients.

## Background

Magnetic resonance imaging (MRI) T2-star (T2*) is a noninvasive method that has been adopted in clinics to quantify tissue iron accumulation. The amount of iron stored in organ tissue is directly proportional to the rate of relaxation time in MRI (1/T2*), called R2-star (R2*) [1–3]. The relationship between R2* evaluated from MRI and liver iron concentration (LIC) assessed by biopsy has been proposed [4–7]. The R2* values can be converted easily to the LIC map. Then, they are used to determine a suitable dose of chelator [8, 9]. Therefore, reliable and precise measurement methods for evaluating R2* are very important in monitoring iron chelation therapy. The R2* can be calculated by fitting data between signal intensities and echo times (TE). Difference curve fitting models yield different results. There are three models for fitting the R2*: mono-exponential, bi-exponential, and mono-exponential with constant-offset (offset) model. Some previous works [6, 8, 10–18] demonstrated that the lowest levels of intra- and inter-reader variability can be obtained when the pixel-wise method was fitted for R2* calculation in an ROI encompassing the whole liver by the offset model. Furthermore, previous works [5, 7] proposed that the vessel pixels in liver parenchyma should be removed to reduce the LIC variation. Therefore, the processes for estimating liver LIC from R2* are as follows: 1. manually defined an ROI to exclude non-body part; 2. calculated R2* by pixel-wise method with offset model and conversion to LIC map; 3. manually defined whole liver ROI; 4. manually excluded vessel from liver parenchyma; and 5. LIC report generator. Some previous works [18–20] proposed the methods for automatically excluding main vessels in user-defined liver region or automated on step number 4. Thus, there are two steps (step 1 and 3) that need to be automated before the whole process of LIC calculation can be performed automatically.

Many automatic segmentation methods of medical images have been proposed. The methods include thresholding [21, 22], watershed [23, 24], random walk [25], active contour models [25–27], statistical shape model [22], level-set [28, 29], graph cuts [22, 30–34], deformable models [35], region growing [29, 36, 37], and deep learning (DL) [33, 34, 38–41]. Regarding semi-automatic segmentation, human intervention is needed, such as manual arbitrary selection of the ROI, initialization of a seed point for region growing or level sets, contour for an active contour model or Laplacian mesh optimization, and seed nodes for random walk [28, 30, 36]. Fully automatic segmentation does not need human intervention. Some human interventions have improved the semi-automatic method by developing algorithms for the automated initialization process [37, 42].

DL is a hot issue for many tasks, including automated liver segmentation techniques. Most of them focus on CT images. Hoang et al. [38], investigated three well-known convolutional neural networks (CNNs), including FCN-CRF, DRIU, and V-net, for liver segmentation in CT abdominal images. For 3D liver segmentation, Lu et al. [34], applied 3D deep CNN to detect and segment the liver simultaneously in contrast-enhanced CT volumes. Hu et al. [39], trained a deep 3D CNN to learn a subject-specific probability map of the liver which identified the initial surface and acted as a shape before the globally optimized surface evolution model. For MR images, Wang et al. [40] trained a 2D U-Net CNN for liver segmentation using abdominal CT and MRI exams. Liu et al. [41] applied a batch normalized U-Net with variable input width to incorporate multiple echoes for liver and vessel segmentation in liver iron quantification. DL is a high-performance technique for object segmentation tasks, but it is a supervised learning technique that requires a large quantity of data and time to train the model for optimum performance.

Another popular segmentation technique is fuzzy c-means (FCM) clustering [43, 44], as well as its modified techniques for improving performance. Some previous works have tried to apply FCM clustering for segmenting various organs in medical images. Wang et al. [45] proposed an adaptive spatial information theoretic FCM clustering to improve the robustness of the conventional FCM for MRI brain segmentation. Mekhmoukh et al. [46] applied particle swarm optimization and level set methods for optimizing the initialization of cluster centers and rejecting outliers for FCM clustering to segment MR brain images. Rundo et al. [47] presented a two-stage computational framework based on FCM clustering for automated sub-segmentation of three tissue types in CT images: cystic/necrotic, calcified, and soft tissue. Some works have tried to apply FCM clustering for liver segmentation, but most of them focused on CT images [48–51]. For MR images, Feng et al. [19], applied FCM clustering to automatically segment the parenchyma and non-parenchyma based on R2* values in manually drawn liver regions. Saiviroonporn et al. [18, 20] applied 2D-FCM clustering that used TE images and LIC maps as the input to segment the vessels from parenchyma inside user-defined ROIs. To automate the whole process of LIC calculation as previous mentioned, the user-defined body-selection and liver ROI steps (step 1 and 3) should be replaced by an automated method.

In this study, we investigated the automated liver segmentation method in the LIC map to develop the automated processes of LIC calculation (for example, automation of step 1 and 3). Based on the above literature reviews, FCM is an efficient algorithm for the segmentation of the internal organs in medical images. Therefore, FCM clustering combined with the anatomical landmark data technique was proposed. We hypothesized that anatomical landmark data should be able to improve the segmentation results from FCM clustering by rejecting unwanted regions that had the same LIC values as the liver region.

## Materials and methods

### Subjects

This study was approved by the review board of Faculty of Medicine, Siriraj hospital, (Si 465/2018), and informed consent was obtained from all of the participants before the research started. A total of 553 MR examinations from 471 thalassemia major patients (139 males and 332 females; aged 21.7 ± 11.6 years) were performed from 2009 to 2015 and were included in this work. The data were separated randomly into 2 groups: the training and testing cohorts, which comprised 80% and 20% of the data (441 and 112 exams), respectively. The training cohort was used to find the suitable segmentation parameters. The testing cohort was used to validate this experiment.

### Liver scan protocols and LIC map calculation

The liver MR images were acquired on a Philips Achieva-XR 1.5 T scanner at a trans-axial mid-hepatic slice. A breath-hold, multi-echo gradient echo sequence was used with the following acquisition protocol: 20° flip angle, repetition time of 80 ms, 20 TEs (1.07–16.27 ms in 0.80 ms increments) or 20 TE images, slice thickness of 10 mm, matrix of 256 × 256 pixels, field of view of 40 cm, and yielding in-plane resolution of 1.6 × 3.1 mm^2^.

The R2* measurement results were transformed to LIC values based on reports from previous works [5–7]. The R2* estimation was performed using the pixel-wise method fitted by the offset model. The LIC map was calculated from R2* using Eq. (1) [5, 9]:

1 $${\text{LIC }} = \, 0.{2}0{2 } + \, 0.0{254} \times {\text{R2}}*$$

The LIC map was used as an input for segmentation processes in this study.

### Manual segmentation

To measure the segmentation performance, the ground truth in our experiments were manually segmented case by case in LIC maps by a board-certified radiologist. The expert manually segmented the liver regions into 2 types in each exam. The first one was the entire liver region. It was used to optimize the membership threshold (MT). The second one was the liver region used in clinical application. It was often segmented smaller than its actual size to avoid uncertainty occurring at the edge of the liver region. The second type was used to optimize the erosion size (ES) for eroding the liver region to be suitable for clinical application.

### Proposed segmentation method

**Fig. 1:**
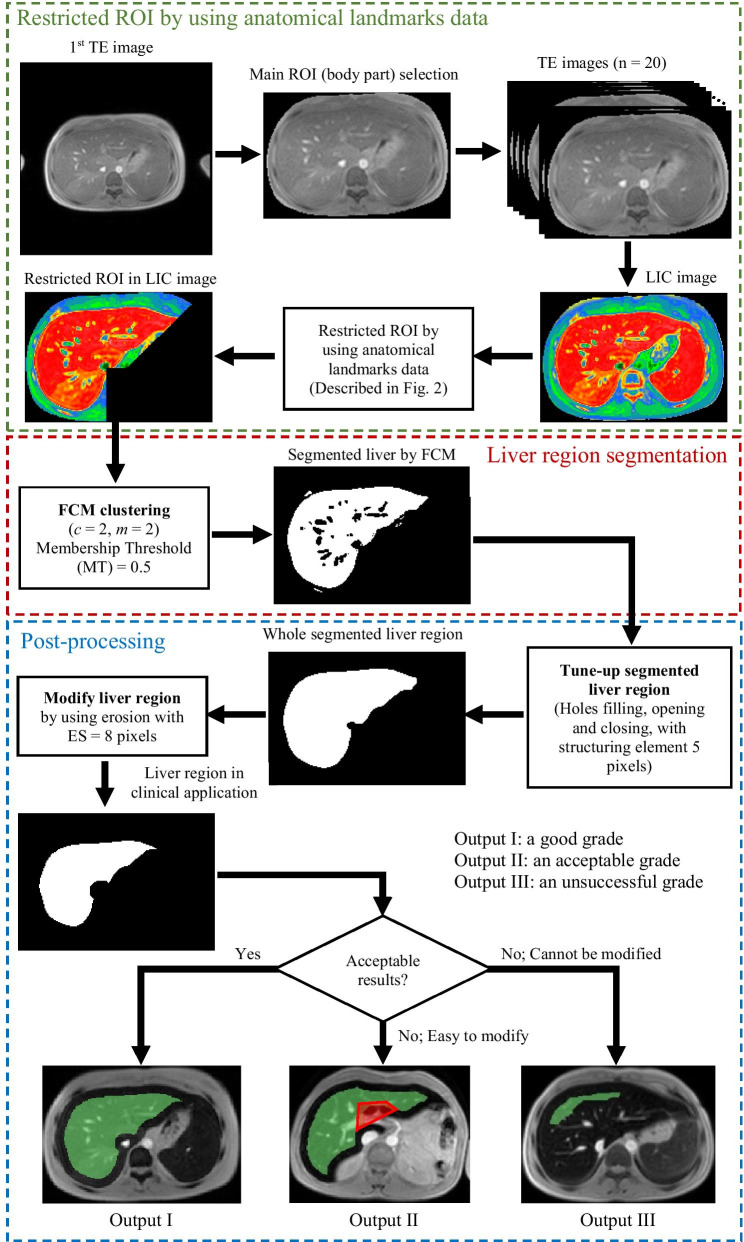
Diagram for applying the proposed method to practical implementation

MATLAB Toolboxes (MathWorks, Inc., Natick, MA, USA) were used for all analytical operations. An overview of the proposed method for liver segmentation is shown in Fig. 1. The method consists of 4 main procedures: main ROI selection, anatomical landmark-based ROI restriction, liver region segmentation by FCM clustering, and post-processing by using mathematical morphologies.

### Main ROI (body part) selection process

**Fig. 2:**
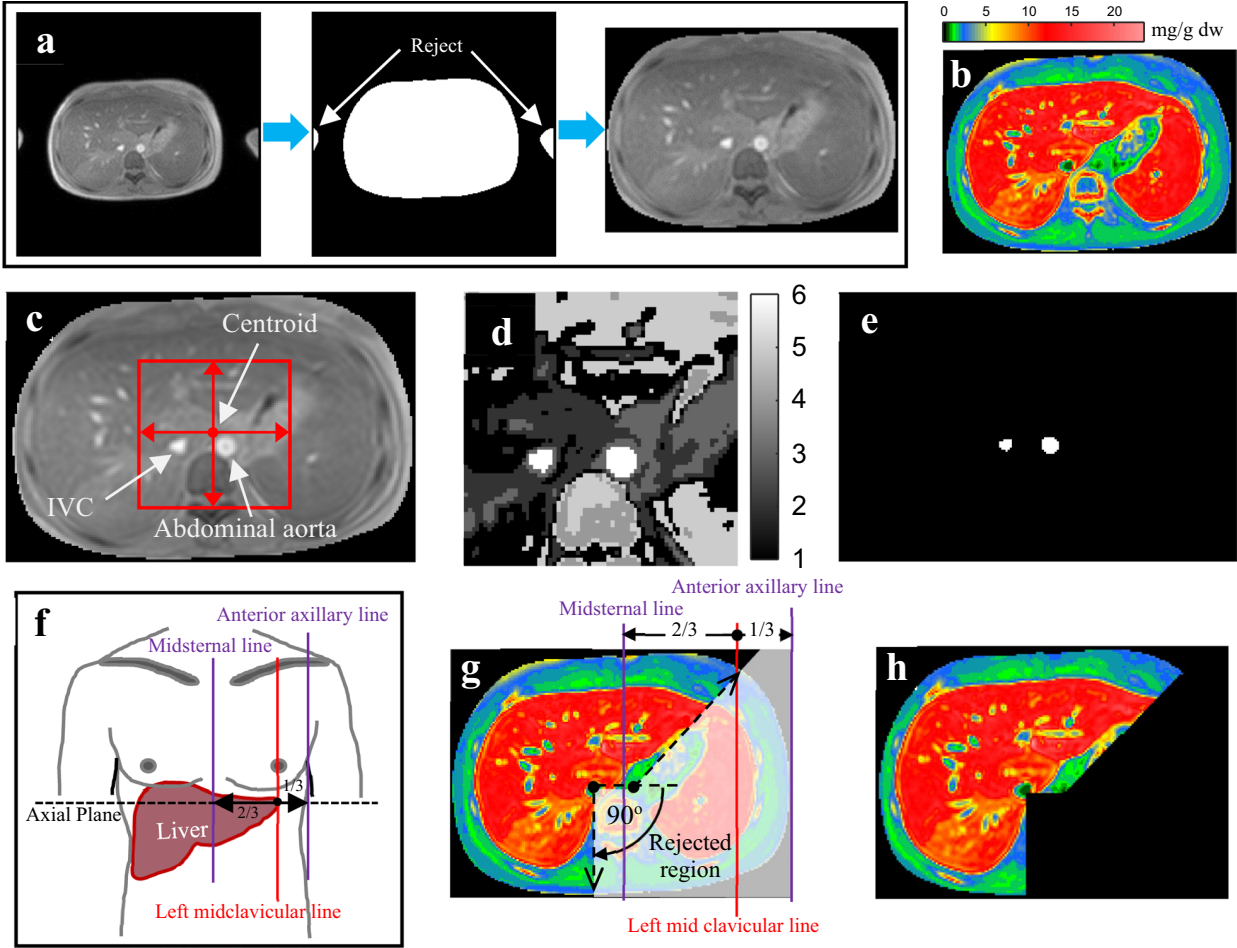
The ROI restricted processes, **a** the main ROI in the TE image, **b** LIC map, **c** the created sub-image including the IVC and abdominal aorta, **d** the segmented image in multi regions, **e** the segmented IVC and abdominal aorta in a normal image size, **f** simple structure of the human, including longitudinal, lines used in this work, **g** the rejected region, and **h** the LIC map in the restricted ROI

In this process, global thresholding by Otsu’s method [52] was applied with the first TE image for separating the background and rejecting excess objects away from the body part. It also reduced calculation time in the liver segmentation process. The biggest object which passed hole-filling algorithm [53] was selected as the main ROI which was used in the next process. Figure 2a shows the result of this procedure.

### Anatomical landmark-based ROI restriction

This procedure was developed because, in some cases, the LIC values in the liver region were in a similar signal as those in adjacent organs, as shown in Fig. 1. Because the segmentation results might include an unwanted region, the ROI for the segmentation process should be restricted by using anatomical landmark data. The procedures for ROI restriction (Fig. 2) are:

1. The main ROI (body part) in all TE images was used to calculate R2* and transform it to the LIC map by Eq. (1), as shown in Fig. 2b.
2. The centroid of the object of interest in the main ROI selection process was calculated. A sub-image was created in the first TE image by expanding the width of the main ROI in 4 directions, with a distance of 20% (by experiment) from the centroid calculated from main ROI to include inferior vena cava (IVC) and abdominal aorta in the sub-image, as shown in Fig. 2c.
3. The IVC and abdominal aorta were segmented in the sub-window by using FCM clustering [43, 44]. The input of FCM consisted of signal intensity values from each pixel. The number of clusters was defined by varying it from 2 to 8 and recording the achievements to detect the IVC and abdominal aorta in the training cohort data; a fuzzy factor of 2 was used [57]. Figure 2d shows the segmented image in multiple regions. The IVC and abdominal aorta in the images typically had the highest intensity values. Therefore, the data in the cluster that had the highest value of centroid were selected as the IVC and abdominal aorta. The IVC and abdominal aorta were placed back in the normal size image as shown in Fig. 2e.
4. The centroids of the IVC and abdominal aorta were calculated and used as the center for creating the rejected region. By anatomical theories, the left midclavicular line is longitudinal and passes through the middle of the clavicle. It usually passes near the nipple, as shown in Fig. 2f and is located to the right of 2 in 3 parts from a midsternal line or to the left of 1 in 3 parts from an anterior axillary line. The liver typically extends from the fifth intercostal space to the right costal margin in the midclavicular line [54–56]. This was used to find the landmarks for rejecting an unwanted region. In MRI, the assumed left midclavicular line was drawn between the middle line of the body region (assumed to be a midsternal line) and the edge of the body region (assumed to be an anterior axillary line). The diagonal dashed line was drawn from the abdominal aorta through the intersection between the left midclavicular line and the edge of the body. The longitudinal dashed line covers the right lobe of the liver, as shown in Fig. 2g. Figure 2h shows the restricted ROI in the LIC image, which is used in the segmentation procedure.

### Liver region segmentation

FCM clustering was used again in this procedure to separate the liver area (object) from others (background). In this procedure, LIC values in the restricted ROI were the input of FCM. To establish the FCM parameters, a fuzzy factor was defined as 2, as before, and the number of clusters was set as 2. A cluster that had a higher centroid value was defined as the liver region and the other one as other organs or background. To optimize the segmentation performance, the membership threshold (MT) value was adjusted from 0.1 to 0.9 (at increments of 0.1).

### Post-processing

After the segmentation process, some segmented images contained various imperfections. The liver regions were disturbed in a binary image by noises and errors. Mathematical morphologies [53, 58], opening and closing with the optimized size of disk shape structure, and hole-filling, were used to proceed to the goal of removing imperfections by accounting for the form and structure of the image. This optimized size of disk shape structure was 5 pixels. It was obtained from the experiments, varying the values from 3 to 7 pixels (data not shown). After that, the biggest object was selected as the liver region.

### Segmentation for clinical application

According to the clinical application, the liver region was often manually segmented smaller than its actual size to avoid uncertainty occurring at the edge of the liver region. For this reason, an erosion algorithm using disk shape structure called erosion size (ES), was varied from 2 to 12 pixels (at increments of 1 pixels) to evaluate the best segmentation performance.

The output results from the proposed method were divided into three categories: a good grade, an acceptable grade, and an unsuccessful grade. A good grade was defined as a completely segmented liver region as shown in Output I in Fig. 1. An acceptable grade was the reasonably well-segmented liver region. The segmentation results could be easily modified by using a simple step as shown in Output II in Fig. 1. The last one was an unsuccessful grade; the segmentation results could not be accepted and was defined as Output III in Fig. 1. They required a full manual segmentation to complete a task.

### Evaluation metrics

To measure the performance of the proposed algorithm, the segmentation results were compared with manual segmentation performed by a board-certified radiologist. Dice similarity coefficient (*DSC*) [59], Jaccard index (*JC*) [60], and Hausdorff distance (*H*) [61] were used to evaluate the segmentation performance. In this work, *DCS* and *JC* were reported in percent (defined as *%DSC* and *%JC*) to consider the differences of the experiment results in more detail. These metrics are calculated as follows:

Let *A* and *B* represent 2 binary regions (the manual and the automatic segmentation); the *%DSC* and %*JC* between them are defined as

2 $$\% DSC \, = \, \frac{{2 \times Area\left( {A \cap B} \right)}}{Area\left( A \right) + Area\left( B \right)} \times 100,$$

and

3 $$\% JC \, = \, \frac{{Area\left( {A \cap B} \right)}}{{Area\left( {A \cup B} \right)}} \times 100.$$

The Hausdorff distance (*H*) is calculated by

4 $$H \, = \, \max \{ \mathop {\sup }\limits_{a \in A} \mathop {\inf }\limits_{b \in B} d(a,b),\mathop {\sup }\limits_{b \in B} \mathop {\inf }\limits_{a \in A} d(a,b)\} ,$$

where *d* is the Euclidean distance and (*a*,*b*) is two points of both contours of *A* and *B*. Perfect segmentation occurs when the *DSC* is one (*%DSC* = 100), *JC* is one (*%JC* = 100), and *H* is zero.

In clinical application, the liver region usually is selected as smaller than the real liver region. ES in post-processing should be optimized as mentioned above. Therefore, the percent false positive (*%FP*) and percent false negative (*%FN*) calculated by (5) and (6) were considered in this situation.

5 $${\text{\% FP }} = \frac{{\begin{array}{*{20}c} {{\text{No}}{\text{. of pixels auto segmented by an algorithm that }}} \\ {\text{shrink more than manually segmented by an expert}} \\ \end{array} }}{{\text{Total amount of pixels manually segmented by an expert}}} \times 100,$$

6 $${\text{\% FN }} = \frac{{\begin{array}{*{20}c} {{\text{No}}{\text{. of pixels auto segmented by an algorithm that}}} \\ {\text{protrude more than manually segmented by an expert}} \\ \end{array} }}{{\text{Total amount of pixels manually segmented by an expert}}} \times 100.$$

Generally, a good segmentation occurs when *%FP* and *%FN* are close to zeros. If *%FP* is too high, under-segmentation occurs. On the other hand, if *%FN* is too high, over-segmentation occurs. In this study, *%FN* was used as the primary metric for determining outcomes. It was calculated as two types, *%FN-type-I* and *%FN-type-II*. The *%FN-type-I* was calculated from the segmented liver region used in clinical application from both automated and manual methods. For *%FN-type-II*, the automated method used the segmented liver region for the clinical application while the manual method used the segmented real liver region. It was used to examine whether the segmentation results were acceptable in practical implementation. They were accepted if the *%FN-type-II* value was zero even if *%FP* was nonzero. It meant that the segmented liver region from the proposed method was not larger than the actual liver region.

### Statistical analysis

The paired Student *t* test was used to examine the difference, such as the difference in segmentation results between conventional FCM clustering method and FCM combined with anatomical landmark data method, which were considered statistically significant when *p* < 0.05.

The scatter and Bland–Altman plots with 95% confidence interval between the median LIC value of the manual and automated methods were analyzed. First, they were used to verify that anatomical landmark data could improve the correlation and agreement between manual and automated methods. Next, they were used to verify that the proposed method could be used in the routine clinical application. The calculated median LIC from the proposed method should be similar to the traditional method (manual method).

## Results

### Segmentation performance

#### Liver region segmentation

The automated segmentation time in each case was 0.31 s on average via PC (Intel Corei7 4.30 GHz, 16 GB RAM) by using the proposed method. The real liver region segmentation was approximately 20 s and 10 s for the clinical application by using the manual tracing method. Therefore, the automated segmentation improved the processing time.

**Fig. 3:**
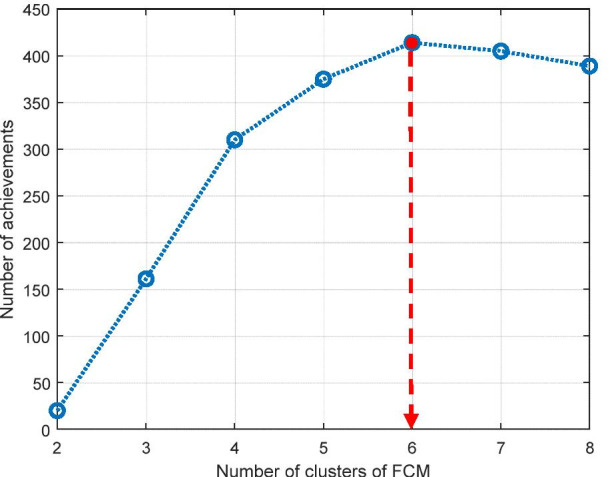
The plot for finding achievement of the IVC and abdominal aorta positions detection

The Main ROI (body part) was segmented successfully in every case. In step (4) in the anatomical landmark data detection process, the number of clusters of FCM was optimized by the experiment as shown in Fig. 3. The maximum number of achievements for detecting the positions of the IVC and abdominal aorta was found when the number of clusters of FCM was 6 clusters.

Table 1 shows the segmentation results from the training cohort by varying MT. The best result by FCM clustering for segmenting the liver region was achieved when MT was 0.8 (*%DSC* = 89.66 ± 13.08, *%JC* = 82.99 ± 14.97, and *H* = 40.16 ± 28.00 mm). After the anatomical landmark data was combined with FCM, the best segmentation result was improved significantly (*p* < 0.001). The *%DSC*, *%JC*, and* H* were 93.33 ± 4.97, 87.83 ± 7.36, and 29.67 ± 20.28 mm, respectively. They were achieved when MT was 0.5. Therefore, MT = 0.5 was an optimal parameter in this study.

**Table 1:** The whole liver region segmentation results in training cohort using only FCM and FCM combined with anatomical landmark data

MT	FCM			FCM combined with anatomical landmark data		
	*%DSC*	*%JC*	*H* (mm)	*%DSC*	*%JC*	*H* (mm)
0.1	85.62 ± 12.87	76.56 ± 15.70	51.66 ± 31.55	92.60 ± 4.35	86.49 ± 6.70	28.83 ± 19.02
0.2	87.15 ± 12.84	78.92 ± 15.45	47.53 ± 31.15	92.97 ± 4.44	87.15 ± 6.79	29.32 ± 19.82
0.3	87.93 ± 13.19	80.23 ± 15.51	44.63 ± 30.76	93.14 ± 4.75	87.48 ± 7.13	29.16 ± 20.09
0.4	88.62 ± 13.05	81.30 ± 15.23	42.55 ± 30.32	93.25 ± 4.89	87.69 ± 7.29	29.40 ± 20.33
0.5	89.08 ± 12.99	82.01 ± 15.07	41.35 ± 29.28	**93.33 ± 4.97**	**87.83 ± 7.36**	**29.67 ± 20.28**
0.6	89.32 ± 13.04	82.43 ± 15.11	40.76 ± 29.14	93.28 ± 5.28	87.79 ± 7.72	30.24 ± 20.73
0.7	89.60 ± 12.95	82.85 ± 14.91	40.07 ± 28.25	93.22 ± 5.41	87.70 ± 7.87	30.93 ± 20.76
0.8	**89.66 ± 13.08**	**82.99 ± 14.97**	**40.16 ± 27.99**	93.02 ± 5.99	87.43 ± 8.48	31.92 ± 21.48
0.9	89.55 ± 13.55	82.92 ± 15.43	39.56 ± 27.26	92.62 ± 6.71	86.83 ± 9.26	33.19 ± 22.08

**Fig. 4:**
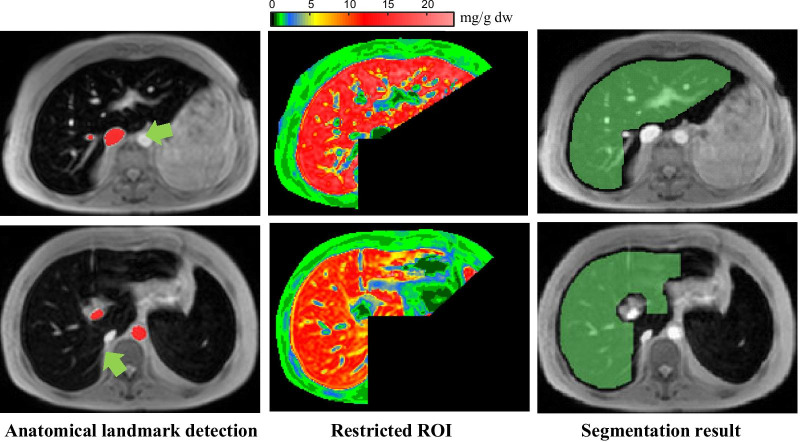
The examples of the failure to detect anatomical landmark data

Table 2 shows that anatomical landmark data could improve the segmentation performance. The number of cases in which the anatomical landmark method could significantly (*p* < 0.001) improve segmentation results was 138 cases (31.29% of all data in training cohort), while the rest were not significantly different (*p* = 0.9707). The averages of *%DSC*, *%JC*, and *H* from the improved cases were 93, 88, and 25 mm compared to 82, 72, and 63 mm from the FCM method alone and obtained the same results of 93, 88, and 31 mm, respectively, in the unimproved cases. The anatomical landmark method identified both vessels correctly in most of the cases (545 of 553 or 98%). Figure 4 shows two examples of the misclassification of the aorta and the IVC in the first and second row, respectively. Although the inaccurate detection occurred as mentioned before, the segmentation processes still carried on. The average of *%DSC*, *%JC* and *H* of these cases were 89.45 ± 2.53, 80.99 ± 4.07, and 41.93 ± 19.77 mm, respectively. They showed that this error had little effect on the segmentation process.

**Table 2:** The whole liver region segmentation results in training cohort by separating the cases on which anatomical landmark data either had effect and no effect on the segmentation accuracy

The number of cases	Evaluation matrices	FCM	FCM combined with anatomical landmark data
Effect: 138 cases	*%DSC*	81.89 ± 18.75	93.55 ± 2.54
	*%JC*	72.16 ± 18.30	87.99 ± 4.37
	*H* (mm)	62.86 ± 27.34	24.79 ± 15.34
Non-effect: 303 cases	*%DSC*	93.20 ± 7.04	93.22 ± 5.75
	*%JC*	87.92 ± 9.84	87.75 ± 8.38
	*H* (mm)	29.83 ± 21.45	31.89 ± 21.84

The FCM combined with anatomical landmark data using MT = 0.5 was applied to the testing cohort. The experimental results were consistent. The *%DSC*, *%JC*, and* H* were 90.10 ± 15.41, 84.51 ± 18.38, and 33.33 ± 34.76 mm, respectively. The segmentation results improved significantly (*p* < 0.001) compared with the results from conventional FCM clustering (*%DSC* = 84.02 ± 25.89, *%JC* = 78.25 ± 26.68, and *H* = 33.33 ± 34.76 mm).

#### Segmentation for clinical application

An optimal parameter from the previous experiment (MT = 0.5) was, then, used in this experiment. The ES was varied to make the liver region smaller in a routine clinical task. The experimental results are shown Table 3. The best *%DSC*, *%JC*, and *H* were 89.87 ± 6.11, 82.06 ± 8.33, and 19.05 ± 13.35 mm, and *%FP*, *%FN-type-I* and *%FN-type-II* were 10.23 ± 10.11, 9.45 ± 7.26, and 0.16 ± 0.61, respectively. These results were obtained when using ES equal to 8 pixels. Therefore, the optimal ES = 8 was used for the testing cohort.

**Table 3:** The segmentation results for the clinical application in training cohort

ES (pixels)	*%DSC*	*%JC*	*H* (mm)	*%FP*	*%FN* *-Type-I*	*%FN* *-Type-II*
2	81.02 ± 5.76	68.49 ± 8.10	23.64 ± 11.33	2.39 ± 6.42	44.06 ± 16.54	3.58 ± 3.29
3	82.36 ± 5.79	70.42 ± 8.26	23.00 ± 11.66	2.68 ± 6.70	39.63 ± 15.76	2.55 ± 2.77
4	83.84 ± 5.82	72.59 ± 8.40	22.30 ± 11.82	3.00 ± 7.04	34.90 ± 14.83	1.72 ± 2.22
5	86.46 ± 5.79	76.57 ± 8.44	21.20 ± 12.29	3.97 ± 7.75	26.36 ± 12.95	0.89 ± 1.58
6	88.58 ± 5.71	79.91 ± 8.21	20.30 ± 12.75	5.64 ± 8.57	18.65 ± 10.82	0.46 ± 1.11
7	89.70 ± 5.82	81.75 ± 8.07	19.65 ± 13.11	8.41 ± 9.52	12.25 ± 8.51	0.24 ± 0.77
8	**89.87 ± 6.11**	**82.06 ± 8.33**	**19.06 ± 13.35**	**10.23 ± 10.11**	**9.45 ± 7.26**	**0.16 ± 0.61**
9	88.98 ± 7.03	80.73 ± 9.37	19.00 ± 13.81	14.84 ± 11.14	5.38 ± 5.11	0.09 ± 0.43
10	86.58 ± 8.21	77.10 ± 10.67	20.33 ± 14.41	20.64 ± 11.93	2.79 ± 3.39	0.05 ± 0.28
11	84.90 ± 8.84	74.61 ± 11.26	21.10 ± 14.40	23.84 ± 12.23	1.94 ± 2.71	0.04 ± 0.23
12	80.71 ± 9.86	68.62 ± 11.94	23.34 ± 14.59	30.66 ± 12.47	0.94 ± 1.76	0.02 ± 0.16

The optimal parameters (MT = 0.5 and ES = 8) from the experiment in the training cohort were applied to the testing cohort. The *%DSC*, *%JC*, and *H* were 89.18 ± 7.70, 81.18 ± 10.43, and 20.45 ± 18.76 mm, and *%FP*, *%FN-type-I* and *%FN-type-II* were 10.23 ± 12.58, 10.54 ± 7.57, and 0.33 ± 1.00, respectively. They represented that the proposed method provided consistent segmentation results in clinical application.

#### Output categories

**Fig. 5:**
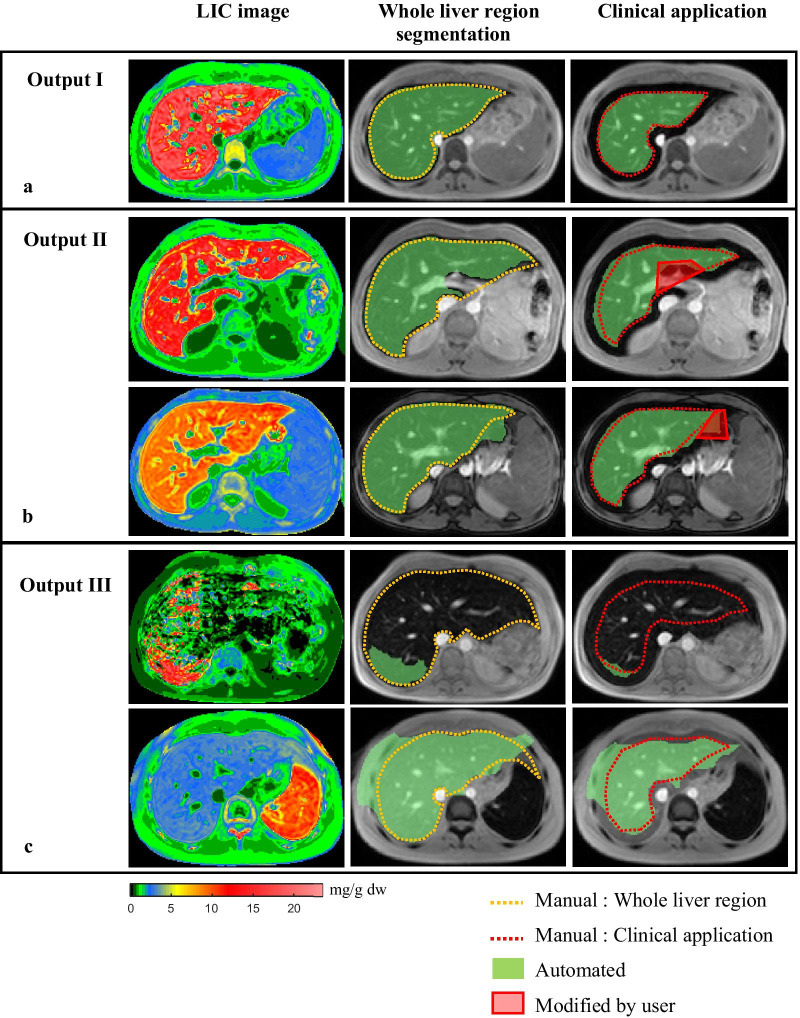
The example of segmentation results in each category from five patients, **a** Output I, **b** Output II, and **c** Output III

Table 4 shows the number of cases of segmentation results for clinical application in each category. The number of Output I, II, and III were about 81%, 11%, and 8% of all data, respectively. A good segmentation result, Output I, is shown in Fig. 5a. The *%DSC*, *%JC*, and *H* of this category in the training and testing cohorts were approximately 91%, 84%, and 15 mm, respectively. The number of an acceptable segmentation results, Output II, which required an easy correction from the user, was approximately 11% of all data, as shown as the red polygons in Fig. 5b. The *%DSC* and *%JC* of this category were more than 85% and 75%, respectively, while *H* was in the range of 28–32 mm. Finally, the remainder, less than 8% of all data (Output III) is shown in Fig. 5c. The segmentation results of Output III failed and could not be easily modified. Their evaluation matrices resulted in unacceptable segmentation performance (*%DSC* < 55, *%JC* < 43, and *H* > 70 mm). Therefore, this category required full manual segmentation by a user.

**Table 4:** The number of cases and evaluation matrices of segmentation results for clinical application in each category

Output categories	FCM combined with anatomical landmark data			FCM
	Training cohort	Testing cohort	All data	All data
Output I				
No. cases	359 (81.41%)	87 (77.68%)	446 (80.65%)	349 (63.11%)
*%DSC*	91.30 ± 3.05	91.70 ± 2.55		
*%JC*	84.13 ± 5.04	84.78 ± 4.30		
*H* (mm)	15.75 ± 9.66	15.38 ± 9.25		
*%FP*	8.83 ± 6.96	6.82 ± 5.33		
*%FN-Type I*	8.47 ± 7.20	10.02 ± 6.98		
*%FN-Type II*	0.00	0.00		
Output II				
No. cases	51 (11.56%)	12 (10.71%)	63 (11.39%)	150 (27.12%)
*%DSC*	86.43 ± 6.49	85.44 ± 7.14		
*%JC*	76.63 ± 9.45	75.18 ± 10.50		
*H* (mm)	32.50 ± 14.77	28.76 ± 14.68		
*%FP*	14.13 ± 12.94	18.00 ± 12.35		
*%FN-Type I*	11.84 ± 7.49	9.01 ± 5.55		
*%FN-Type II*	0.59 ± 0.81	1.09 ± 1.79		
Output III				
No. cases	31 (7.03%)	13 (11.61%)	44 (7.96%)	54 (9.76%)
*%DSC*	54.26 ± 30.74	47.31 ± 23.93		
*%JC*	42.87 ± 27.91	33.85 ± 20.02		
*H* (mm)	72.39 ± 45.58	96.26 ± 46.01		
*%FP*	53.12 ± 32.01	63.14 ± 25.31		
*%FN-Type I*	6.42 ± 6.81	5.28 ± 12.39		
*%FN-Type II*	0.65 ± 1.80	0.37 ± 1.32		
Total	441	112	553	553

For *%FP* and *%FN*, Output I showed that *%FN-type-II* was zero, and *%FP* and *%FN-type-I* were approximately 8%*.* Output II included cases in which the *%FN-type-II* was approximately 0.7%, while the *%FP* and *%FN-type-I* were greater than Output I, approximately 15% and 11%, respectively. In Output III, *%FN-type-II* and *%FN-type-I* were 0.6% and 6% while *%FP* was extreme high, approximately 55%. They clearly showed the under-segmentation.

The quality of segmentation output was improved when using FCM combined with anatomical landmark data as shown in Table 4 (the evaluation matrices of conventional FCM were not shown). The number of Output I was increased from approximately 63–81% of all data. The number of Output II was reduced from approximately 27–11% of all data. Finally, Output III was improved as well. Their number was decreased from approximately 10–8% of all data.

### Comparison between median LIC from automated and manual method

**Fig. 6:**
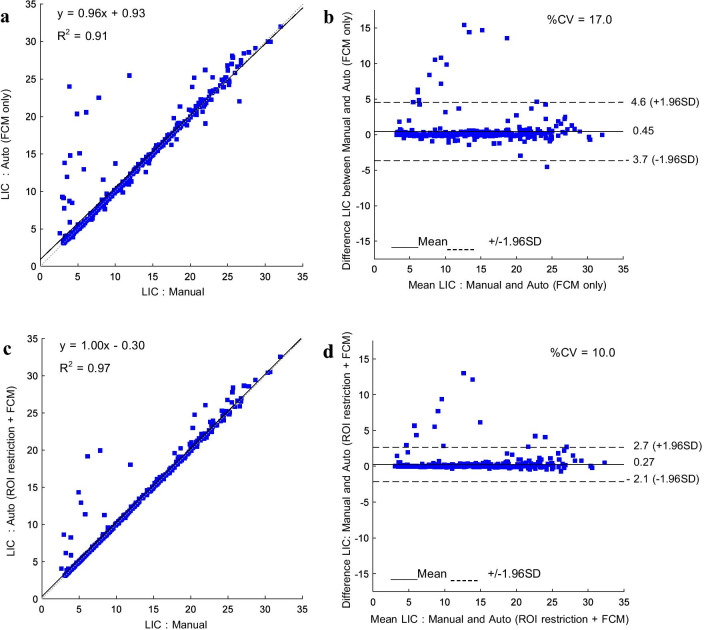
The scatter plot and Bland–Altman plot of median LIC in a comparison between manual and automated methods with 95% confidence intervals, **a** without ROI restriction process and, **b** with ROI restriction process

The scatter and Bland–Altman plots between the median LIC values of the manual and automated segmentation with and without the ROI restriction step for training cohort are shown in Fig. 6. It shows that the ROI restriction step could improve the segmentation performance by considering the better correlation and agreement between manual and automated methods. Figure 6a, c show that R-square (R^2^) was improved from 0.91 to 0.97 and Fig. 6b, d show that the percent coefficient of variation (%CV) was reduced from 17 to 10%.

**Fig. 7:**
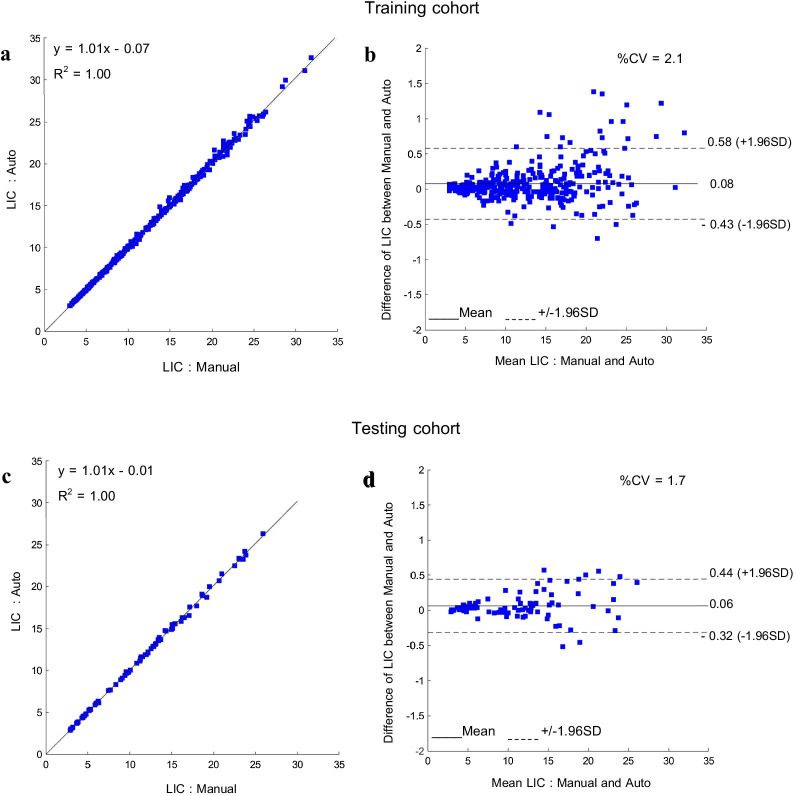
The scatter plot and Bland–Altman plot of median LIC in a comparison between automated and manual methods with 95% confidence intervals, **a**, **b** from the training cohort, **c**, **d** from the testing cohort

Figure 7 shows the scatter and Bland–Altman plots between the median LIC values of the manual and the proposed method. The output II and III were already modified by the user. For the training cohort, in Fig. 7a, b and R^2^ and %CV were 1.00 and 2.1% with an interval of − 0.43%, 0.58%, respectively. For the testing cohort in Fig. 7c, d R^2^ and %CV were 1.00 and 1.7%, respectively, with an interval of − 0.32%, 0.44%. This indicated that the proposed method had excellent correlation and agreement with the manual method.

## Discussion

In this procedure, evaluation of the median LIC in thalassemia major patients during routine clinical tasks required a total of 5 steps, as mentioned in the introduction. Only the user-defined body-selection and liver ROI steps (step 1 and 3) still required user action. To automate the whole process of LIC calculation, steps 1 and 3 need to be automated. In this study, we developed the automated body part selection and liver ROI segmentation to fill this gap. The results showed that our method successfully segmented the liver region with high values of evaluation matrices. We found that our proposed method automatically segmented a good grade output (Output I) in approximately 81% of all data. Approximately 11% of all data were an acceptable grade output (Output II). Only 8% of all data were an unsuccessful grade (Output III). The correlation between median LIC values from our proposed method and the manual method was also high. The average processing time was reduced. It represented that our method could be applied to automate the whole process of LIC calculation in routine clinical application and could reduce the workload of the user.

For main ROI (body part) selection process, the body part was successfully segmented for all of data. We used the classic global thresholding using Otsu’s method, although some of the advanced histogram-based methods for MR images were proposed, such as MedGA [62] and PSOTHE [63]. They applied the enhancement algorithms that helped to improve the quality of images that undergo automated segmentation by using thresholding methods. Because the body’s pixel intensity was clearly different from the background, the basic algorithms, global thresholding method followed by holes filling, could be easily performed. There were only eight cases in which anatomical landmark data failed to be detected. The intensity of the abdominal aorta was lower compared to the surrounding objects, as shown in the examples in Fig. 4. Based on our assumption, the pixel intensities in the abdominal aorta should be higher than others. Therefore, another brighter object (surrounding vessel) was detected, instead, after the clustering process. However, the average evaluation matrices of these cases were also acceptable, and, therefore, had little impact on the segmentation process.

We proposed the anatomical landmark data detection to restrict body ROI in order to reject the unwanted regions (other organs) that connected to the liver and had the same LIC values as the liver; conventional FCM segmented them as the liver region. In the experiment for entire liver region segmentation, the segmentation performance could be significantly improved for more than 30% of all data in the training cohort. This proposed process did not affect the remainder of the examinations. Their segmentation results were still good and not significantly different. As well as the experiment for clinical application, the number of Output I was increased, and the numbers of Output II and Output III were decreased. It showed that this approach improved the segmentation performance based on our assumption. Moreover, Fig. 6 confirmed that the proposed ROI restriction process was a necessary step. Although there was a correlation of median LIC between the manual and both automated methods, only FCM and FCM combined with the anatomical landmark data, were high (R^2^ > 0.9). The agreement was improved obviously (%CV was reduced from 17 to 10%) after applying the ROI restriction process.

**Fig. 8:**
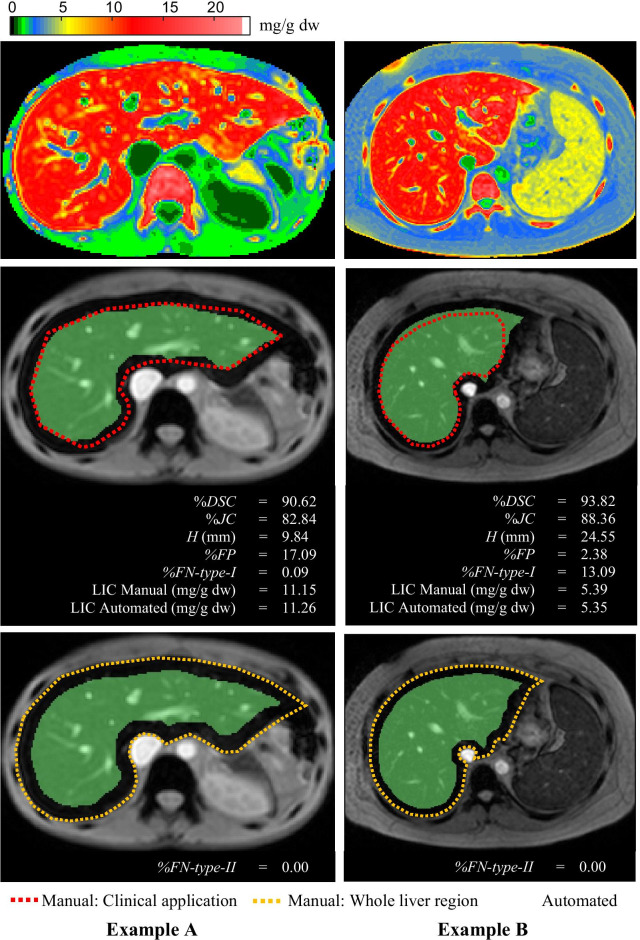
The examples that showed the reason why *H* was not suitable for this study and *%FP*, *%FN-type-I* and *%FN-type-II* should be considered in the experiment for the clinical application

In this study, we focused on the median LIC which was calculated from all the pixels in an ROI. Therefore, the segmentation results should be considered from the perspective of the region. The *%DSC* and *%JC* were calculated from all pixels in an ROI while *H* was calculated from only the contour of an ROI. Therefore, *H* might not be suitable for this study. The example that supported this situation is shown in Fig. 8. Examples A and B were a good grade output (Output I). Example A had a high *%DSC* and *%JC*. The median LIC in the liver region from both methods were approximately the same, less than 1% difference, and *H* was 9.84 mm. All of them showed a good segmentation and a good correlation in median LIC. For Example B, *%DSC* and *%JC* were still high. Likewise, the median LIC in the liver region from both methods were less than 1% different; they presented a good segmentation and a good correlation in median LIC, as well. *H* was more than two times higher than Example A because the distal region of the right lobe of the liver was segmented by the automated method, while it was not done by the manual method. For this reason, *%DSC* and *%JC* were more suitable than *H* for evaluating the segmentation performance in this study.

The reason that *%FP*, *%FN-type-I* and *%FN-type-II* were considered in the experiment for clinical application was they could indicate the under- and over-segmentation, while other evaluation matrices were unable to show these details. Typically, the small values of *%FP* and *%FN-type-I* represented a good overview of segmentation performance, but, in our study, the critical part for the user was to accept the segmented liver ROI when it was only inside the entire liver region *(%FN-type-II* = 0 as in Output I). As shown in Fig. 8, the *%FP* of Example A and *%FN-type-I* of Example B were slightly high, but their *%FN-type-II* was zero. Therefore, they were classified as a good grade output. In the experiment for clinical application, the best segmentation result was selected based on *%DSC* and *%JC*. The *%DSC* and *%JC* were maximal at ES equal to 8 pixels, while *%FN-type-II* was non-zero. When %FN-type-II was non-zero, some of the liver regions segmented by the automated method were larger than the real liver regions; this required user adjustment or Output II. Actually, *%FN-type-II* could be reduced to zero using a larger ES. As shown in Table 3, *%FN-type-II* tended to decrease steadily to zero if the ES was increased continuously. But it was not accepted because it would result in a larger *%FP*, indicating over under-segmentation occurred, which increased cases of Output III. *%FP* of Output I (including Output II after correction) was approximately 8%. Although it represented the under-segmentation, it was not critical. By considering median LIC comparison between 2 methods, %CV was approximately 2% as shown in Fig. 7. It was acceptable, which is similar to a previous report by Saiviroonporn et al. [6]. They reported that the intersite observer variability was approximately 2.5%. These variabilities occurred from slight differences in liver region segmentation. They were little and insignificant errors.

Although the morphological processes were applied to eliminate some errors in the clustering process, the results were still not perfect. There were 2 main reasons for making segmentation errors as in Output II. The first reason involved vessels in the parenchyma that were large in some cases, as shown in the top row of Fig. 5b. Because the pixel values in vessel regions in the LIC map were low, these regions were rejected in the clustering process. Only the small vessels were modified by morphological processes while the large vessels could not be similarly modified. The other reason was shown in the bottom row of Fig. 5b. There was a portion that had the same LIC as the liver that extended from the liver region, so it was over-segmented and required manual elimination. For output III, the first cause of the error was the quality of the LIC map. There were artifacts in liver parenchyma (back and green colors or low LIC values) in an LIC map from patients with a severe iron overload (LIC > 30 mg/g dw), as shown in the top row of Fig. 5c. Consequently, the artifact pixels in the liver parenchyma were considered as the background cluster in the FCM clustering process. The liver region was segmented into small regions which were then eliminated by a post-processing step. The second cause was shown at the bottom row of Fig. 5c, which was the same as the second reason of Output II, but there was more than one protrusion from the liver region that could not be eliminated.

The comparison between the proposed method and the methods of the state-of-the-art on liver MRI segmentations are shown in Table 5. *DSC*, Number of exams, and Run-time obtained by these methods are displayed. Since they had different data sets, experimental settings, and resources, their results presented in Table 5. could not be directly compared. The most comparable is the segmentation performance for the segmented real liver region in each slice (2D segmentation). Although some works proposed 3D segmentation, their segmentation process was performed slice by slice before being put together later. Therefore, the comparison was considered in terms of the average per slice. Because *DSC* was reported in all methods, the *%DSC* from the segmented liver region in our experiment was selected and converted to *DSC* by dividing by 100. Bereciartua et al. [26] used the active contours method that required the initialization by the user. Huynh et al. [42] used the active contours method as well, but the automated initialization step was proposed by using the watershed segmentation to determine the liver candidate region. *DSC* from our method was better than these two methods. Other methods were equivalent to or better than our method by considering *DSC*, but there were some different issues compared to ours. The segmentation procedure in Shen et al. [64] had an image registration process between two images, similar to the method from López-Mir et al. [24], which required information from the previous slice as input for the current slice. Therefore, they required correlation between two or more images and more computational time. Göçeri [65] only used ten slices in his experiments which were likely to affect the robustness of the algorithm when used in the larger data set. Wang et al. [40] and Jansen et al. [66] applied CNNs which required learning time and computational power. Although, our method could not be judged to be better than others, it was a reasonable method both in terms of efficiency and resource utilization. Moreover, the aim of this work focused on median LIC calculation. The experimental results showed that the median LIC from our automated method provided an excellent correlation and agreement with the manual method, as shown in the plots in Fig. 7. It proved that our proposed method could be applied to replace the current ones.

**Table 5:** The comparison of liver segmentation methods

Authors	*DSC*	Number of data	Run time
López-Mir et al.[22]	0.95	16 exams 59–120 slices/exam	7 s/image (Intel Corei5 2.80 GHz CPU, 2 GB RAM)
Bereciartua et al. [24]	0.90	18 exams 21 slices/exam	0.53 s/image (Intel core2quad 3.00 GHz CPU, N/A RAM)
Göçeri [63]	0.95	10 slices	16.80 s/image (Intel Pentium 2.40 GHz CPU, 2 GB RAM)
Shen et al. [62]	0.94	40 exams 44 slices/exam	20 min/case (Intel Corei5 1.3 GHz CPU, 8 GB RAM)
Huynh et al. [61]	0.91	27 exams 44–120 slices/exam	8.4 min/case (Intel Xeon 2.66 GHz CPU, N/A RAM)
Wang et al. [38]	0.93 (T2*w) 0.95 (T1w)	168 exams (T2*w) 6 slices (TEs)/exam 100 exams (T1w)	N/A
Jansen et al. [64]	0.95	55 exams 100 slices/exam 33 exams for training 3 exams for validation 19 exams for testing	N/A
Proposed method	0.93	537 exams 20 slices (TEs)/exam 441 exams for training 112 exams for testing	0.31 s/image (Intel Corei7 4.30 GHz, 16 GB RAM)

The average time for traditional (manual) method was only 10 s for each case and was not difficult to complete. When considering the overall workflow for reporting the median LIC of our method, 81% of data did not require any actions from the user (processing time was approximately 0.3 s); 11% of data needed approximately 2–5 s to modify the results, and only 8% required the manual method. The average of operation time for all categories was 1.5 s. In summary, our proposed method could reduce the average of operation time less than one-fifth compared to the traditional method. Therefore, it streamlined the work and greatly reduced the workload of the users.

There are some limitations in this study. The input of FCM clustering in this study was only LIC images. If the quality of LIC images was not good enough, it would cause poor segmentation performance. The combination of gray values of TE images and LIC values (multi-dimensional FCM), might help in this situation, which was inspired by our previous research [18, 20]. Next, a single membership threshold (MT) value was optimized and used for all data. Similar to [20], we noticed that each LIC range might be suitable for different MT values. Therefore, the adaptive MT value for each LIC range or each LIC image could help to improve the segmentation performance. This revision in our method is planned for our future studies.

## Conclusion

This study aimed to develop an automated liver segmentation in MR images by using the LIC map to automate the whole process for median LIC calculation in clinical application. The FCM clustering technique combined with anatomical landmark data was applied for segmentation processes. Morphological processes were applied in post-processing to decrease the segmentation errors and adjust the liver region for clinical usage. The experimental results showed that the proposed method could increase the efficiency of the conventional FCM clustering. It provided good grade outputs of approximately 81% of all data with good evaluation matrices. Approximately 11% of the total data required an easy modification step to correct the segmentation results. The rest, approximately 8%, needed manual segmentation. A high correlation in the median LIC between our proposed method and the current method was shown in our experiments. Therefore, our method could be used in place of the current method. Although the manual liver segmentation was time-consuming but not complicated, our automated method could reduce the workload of users.

## Data Availability

The data sets analyzed in this study are available from the corresponding author on reasonable request.

## Abbreviations

MR: Magnetic resonance
MRI: Magnetic resonance imaging
TR: Repetition time
TE: Echo time
T2*: T2-star
R2*: R2-star
LIC: Liver iron concentration
ROI: Region of interest
IVC: Inferior vena cava
FCM: Fuzzy c-means clustering
*DSC*: Dice similarity coefficient
*JC*: Jaccard index
*H*: Hausdorff distance
*FP*: False positive
*FN*: False negative
MT: Membership threshold
ES: Erosion size
